# Application of nonnegative matrix factorization to improve profile-profile alignment features for fold recognition and remote homolog detection

**DOI:** 10.1186/1471-2105-9-298

**Published:** 2008-07-01

**Authors:** Inkyung Jung, Jaehyung Lee, Soo-Young Lee, Dongsup Kim

**Affiliations:** 1 Department of Bio and Brain Engineering, KAIST, Daejeon, 305-701, South Korea; 2 KAIST Institute for the BioCentury, KAIST, Daejeon, 305-701, South Korea

## Abstract

**Background:**

Nonnegative matrix factorization (NMF) is a feature extraction method that has the property of intuitive part-based representation of the original features. This unique ability makes NMF a potentially promising method for biological sequence analysis. Here, we apply NMF to fold recognition and remote homolog detection problems. Recent studies have shown that combining support vector machines (SVM) with profile-profile alignments improves performance of fold recognition and remote homolog detection remarkably. However, it is not clear which parts of sequences are essential for the performance improvement.

**Results:**

The performance of fold recognition and remote homolog detection using NMF features is compared to that of the unmodified profile-profile alignment (PPA) features by estimating Receiver Operating Characteristic (ROC) scores. The overall performance is noticeably improved. For fold recognition at the fold level, SVM with NMF features recognize 30% of homolog proteins at > 0.99 ROC scores, while original PPA feature, HHsearch, and PSI-BLAST recognize almost none. For detecting remote homologs that are related at the superfamily level, NMF features also achieve higher performance than the original PPA features. At > 0.90 ROC_50 _scores, 25% of proteins with NMF features correctly detects remotely related proteins, whereas using original PPA features only 1% of proteins detect remote homologs. In addition, we investigate the effect of number of positive training examples and the number of basis vectors on performance improvement. We also analyze the ability of NMF to extract essential features by comparing NMF basis vectors with functionally important sites and structurally conserved regions of proteins. The results show that NMF basis vectors have significant overlap with functional sites from PROSITE and with structurally conserved regions from the multiple structural alignments generated by MUSTANG. The correlation between NMF basis vectors and biologically essential parts of proteins supports our conjecture that NMF basis vectors can explicitly represent important sites of proteins.

**Conclusion:**

The present work demonstrates that applying NMF to profile-profile alignments can reveal essential features of proteins and that these features significantly improve the performance of fold recognition and remote homolog detection.

## Background

Nonnegative matrix factorization (NMF) is a feature extraction method that has a property of intuitive part-based representation of the original feature [1]. Due to the non-negativity constraint, the parts produced by NMF can be interpreted as subsets of elements that tend to occur together in sub-portion of the dataset [2]. In this way, NMF can be applied to the multidimensional dataset in order to discover patterns and to help interpretation of large biological dataset. This unique ability makes NMF a potentially promising method for biological sequence analysis.

Proteins are said to have a common fold if they share a similar spatial arrangement of major secondary structures. Proteins in the same fold may have low sequence similarity, but they often share similar functions. Fold recognition is to detect a group of proteins that share the common fold with a query protein. It can provide valuable information about the functional role and structure of unknown proteins.

In general, there have been two common approaches for remote homolog detection. The first approach is solely based on sequence information, whereas the second approach uses structural information in addition to sequence information. Hidden Markov Model (HMM) method [3], PSI-BLAST [4], FFAS [5], Picasso [6], and COMPASS [7] can be classified into the first method. GenTHREADER [8], 3D-PSSM [9], FUGUE [10], and PROSPECT [11,12] represent the second approach. Currently most remote homolog detection methods are based on profile-profile alignment (PPA). Some examples are SP3 method [13], ProfNet [14], COACH [15], and HMM-HMM comparison method (HHsearch) [16]. Although these methods are reliable for recognizing relatively close homologs related at the family level, there is still difficulty in finding related remote homologs, reaching only 25% sensitivity at 90% specificity at the superfamily level and very low sensitivity at the fold level.

Recently, the introduction of support vector machine (SVM), a machine learning method, brings remarkable performance improvement in remote homolog searches. Examples are SVM-HMMSTR [17], SVM-I-sites [18], SVM-pairwise [19], SVM-Fisher [20], and profile-profile alignment with SVM [21]. More recently, several kernel methods such as local alignment kernels [22], profile-based direct kernels [23] and cluster kernels [24] are developed to derive a more powerful remote homolog detection. Among them, the method based on profile-profile alignment combined with SVM [21] detects 14% of remotely related proteins with 90% specificity at the fold level. Even though previous SVM-based methods have an ability to recognize the essential features from alignments of remotely related proteins, they do not provide the features with intuitive biological meaning. In addition, the dimension of the profile-profile alignment feature vectors for SVM is quite high, considering the number of intrinsic feature vectors for fold recognition. In such cases, the effect, referred to as the curse of dimensionality, may occur and negatively influence the classification of a given data set.

The methods known as feature extraction techniques can be applied to reduce this problem. There are several linear feature extraction techniques, such as principal component analysis (PCA), independent component analysis (ICA), and multidimensional scaling (MDS), as well as nonlinear analysis such as ISOMAP, LLE, and self-organized feature maps. Among them, nonnegative matrix factorization (NMF) is a linear technique that is characterized by its unique ability of intuitive part-based representation of the original feature [1]. In previous studies, NMF is applied to biclustering of gene expression data [25] and discovering semantic features [26]. As an attempt to popularize the NMF method in the biological data analysis community, LS-NMF [27] and bioNMF [2] are developed.

In this paper, we investigate the possibility of applying NMF to the profile-profile alignment features used for fold recognition and remote homolog detection. We expect that NMF would extract essential features from profile-profile alignment (PPA), improving the performance of fold recognition and identifying remote homolog relationship more accurately. The PPA features have two characteristics that are appropriate for utilizing NMF. First, not all PPA features are needed for recognition of each fold. Instead, a small portion of the sequence is usually enough for each decision, while some portions coming from poor alignments or improper profiles may act as "noises." This suggests that NMF can improve the performance of SVM by explicitly using part-based representation of essential features with lower dimensionality. Secondly, the PPA score is essentially the sum of log odds scores, which can be decomposed linearly. The assumption of linear decomposition used in NMF fits well with this characteristic.

## Results

### Performance comparison for fold recognition at the fold level

In this section, we describe the fold recognition performance of SVM with NMF features compared to that of PPA features, along with HHsearch and PSI-BLAST results. In this work, we define the fold recognition problem in most rigorous way in that we only consider the situation where no proteins sharing the same superfamily members with a query protein are in the template library. To validate our method, we create training set (2437 proteins) and testing set (630 proteins and 34 folds) using SCOP 1.67 in such a way that the two sets do not share the same superfamily members.

**Figure 1:**
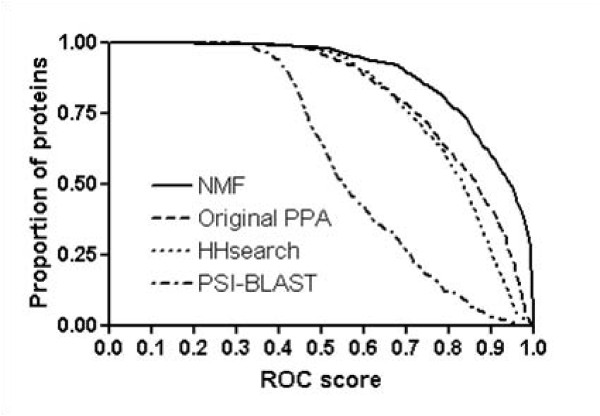
**ROC scores of various methods for fold recognition at the fold level**. The x-axis represents the ROC score and the y-axis represents the proportion of proteins with better performance than the corresponding ROC score. NMF, Original PPA, HHsearch, and PSI-BLAST denote SVM with NMF features, original PPA features, HMM-HMM alignment method, and PSI-BLAST, respectively. The results show that NMF features greatly improve the performance of fold recognition. The mean ROC score of NMF feature is 0.91, while those of original PPA feature, HHsearch, and PSI-BLAST are 0.82, 0.80, and 0.59, respectively.

Figure 1 shows the ROC scores of SVM classifier output, using NMF features and original PPA features. It also shows the results of HHsearch and PSI-BLAST. Clearly, at high level of ROC scores, SVM with NMF features outperforms the original SVM method and the two other popular methods, HHsearch and PSI-BLAST. By using NMF features 30% of testing set correctly recognize homolog proteins with > 0.99 ROC scores, while those of original PPA feature and HHsearch are only 1.5%. In addition, 60% of all proteins in the testing set have ROC score of > 0.9 when using SVM method with NMF features. The corresponding figures are 41%, 26%, and 3% for original PPA features, HHsearch, and PSI-BLAST, respectively.

**Figure 2:**
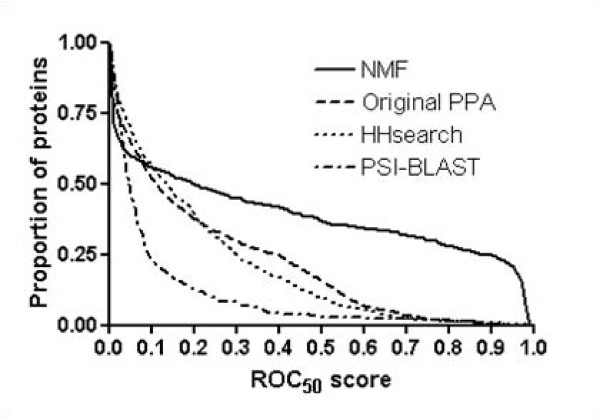
**ROC_50 _scores of various methods for fold recognition at the fold level**. The x-axis represents the ROC_50 _score and the y-axis represents the proportion of proteins with better performance than the corresponding ROC_50 _score. The mean ROC_50 _score using NMF features is 0.44, while the corresponding figures are 0.21, 0.20, and 0.10 for original PPA features, HHsearch, and PSI-BLAST, respectively. At > 0.95 ROC_50 _scores, 22% of protiens with NMF features correctly detect remotely related proteins, whereas original PPA features detect almost none.

In terms of ROC_50 _scores, SVM with NMF features detects homolog proteins at the fold level with 0.44 mean ROC_50 _score. In contrast the cases of original PPA feature, HHsearch, and PSI-BLST achieve only 0.21, 0.20, and 0.10 mean ROC_50 _scores, respectively (figure 2 and Table 1). Furthermore, at ROC_50 _score of > 0.95, NMF features notably improve performance by which 22% of proteins well recognize homolog proteins while original PPA and HHsearch detect almost none. Especially, from the high performance region of ROC_50 _scores for fold recognition, we note that in the view of the performance of fold recognition, high performance region (> 0.75) is more improved than low performance region (< 0.25) by NMF features. NMF improves the performance by roughly fifty folds at ROC score of > 0.90. These results indicate that NMF removes "noises" that may have originated from poor alignments or improper features in the original PPA method, providing enhancement of fold recognition performance.

**Table 1:** The mean ROC_50 _scores at the fold and superfamily level

**Methods**	**Mean ROC_50 _score**	
	**Fold**	**Superfamily**
**NMF**	0.44	0.66
**Original PPA**	0.21	0.49
**HHsearch**	0.20	0.67
**PSI-BLAST**	0.10	0.33

### Performance comparison at the superfamily level (remote homolog detection)

**Figure 3:**
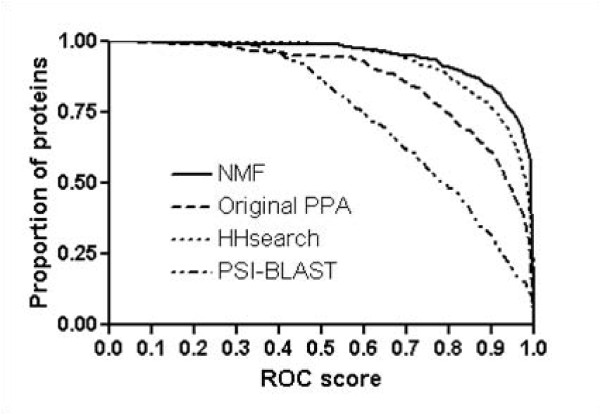
**ROC scores of various methods for remote homolog detection at the superfamily level**. The performance of NMF features is overall better than that of the original PPA features. NMF features achieve over 0.99 ROC scores for 59% of all proteins in the testing set, whereas those of original PPA features, HHsearch, and PSI-BLAST are 30%, 43%, and 11%, respectively. The mean ROC scores of NMF features, original PPA, HHsearch, and PSI-BLAST are 0.95, 0.87, 0.93, and 0.75, respectively.

**Figure 4:**
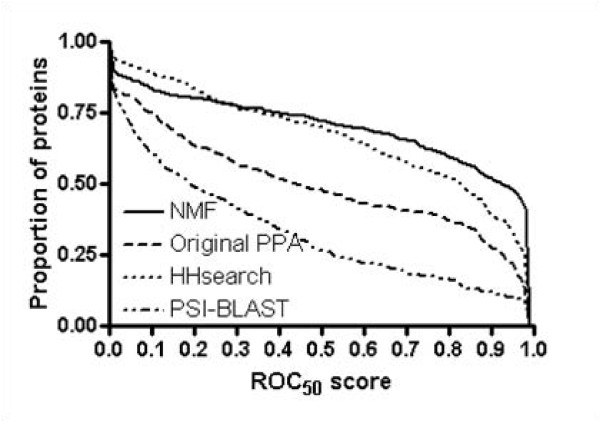
**ROC_50 _scores of various methods for remote homolog detection at the superfamily level**. The mean ROC_50 _score of NMF feature (0.66) is better than those of original PPA (0.49). At > 0.90 ROC_50 _scores, 52% of proteins with NMF features correctly detect remotely related proteins, whereas by using original PPA features only 28% of proteins detect remote homologs and that of HHsearch is 39%.

**Figure 5:**
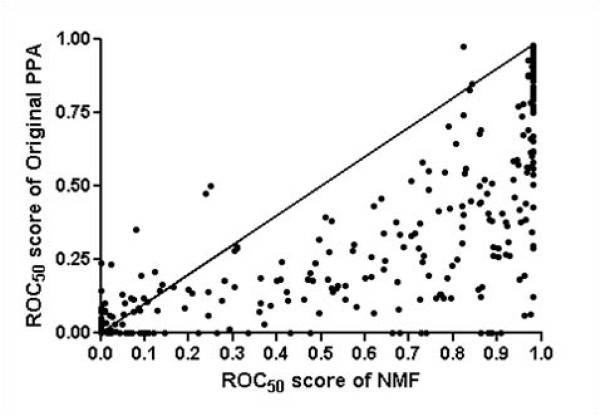
**ROC_50 _score using NMF features versus using original PPA features**. The figure represents ROC_50 _score of NMF features versus that of original PPA features. Applying NMF degrades the performance for only 10% proteins in testing set but increases the performance for more than 70% proteins of testing set.

We also evaluate the performance of NMF features at the superfamily level using ROC and ROC_50 _scores. We construct new training and testing sets using SCOP 1.69, where the testing set (435 proteins and 94 superfamilies) and training set (2342 proteins) do not share the same family members. Figure 3 shows that the performance of NMF features is overall better than that of the original PPA features. With NMF features 59% of all proteins in testing set achieve ROC scores of > 0.99, whereas those of original PPA features, HHsearch, and PSI-BLAST are 30%, 43%, and 11%, respectively. The mean ROC scores of NMF feature, original PPA, HHsearch, and PSI-BLAST are 0.95, 0.87, 0.93, and 0.75, respectively. Additionally, figure 4 indicates that the mean ROC_50 _score of NMF feature (0.66) is significantly improved from those of original PPA features (0.49). At > 0.90 ROC_50 _scores, 52% of proteins with NMF features correctly detect remotely related proteins, whereas 28% and 39% for original PPA features and HHsearch, respectively. Figure 5 shows ROC_50 _score of NMF features versus that of original PPA features. Nearly all points are to the right-hand side, indicating that NMF features effectively improve the performance of remote homolog detection. Applying NMF degrades the performance for only 10% proteins in testing set but increases the performance for more than 70% proteins. Noting that 25% of proteins with ROC_50 _scores < 0.25 in original PPA are significantly improved (> 0.5), we conclude that NMF recognizes essential features well even in the cases where original PPA features are not sufficiently good for detecting remote homologs.

### Benchmarking with LSTM, LA-kernel, and SW-PSSM at the fold and superfamily level

Recently, several kernel-based methods such as LA-kernel [22] and SW-PSSM [23], and a model-based method such as LSTM [22,28] have been developed. Here, we compare our methods with those methods. However, there are some problems that make the direct comparison difficult. First, datasets are different; datasets for our method is based on SCOP 1.67, while the other methods rely on SCOP 1.53 to create datasets. Fortunately, programs for LA-kernel, LSTM, and SW-PSSM are available, and can be easily trained and tested on our datasets. However, there is another problem that the meaning of the score of our method is different from that of the other methods; kernel-based methods, and LSTM give a score measuring that a certain protein may belong to a specific superfamily (or fold), while our method gives a score measuring that two proteins may belong to the same superfamily (or fold). Therefore, to compare the methods, we need to create some sort of scheme that converts our scores to scores that have the same meaning with those of kernel-based methods. In fact, it is possible to develop a highly elaborated scheme, for example, a kernel-based method. However, in this work, we use the simplest scheme; we simply calculate the mean value of SVM output scores of all templates in a specific superfamily (or fold). In addition, training and testing procedure is different. Nonetheless, we measure the ROC and ROC_50 _scores for the present method, LSTM, LA-kernel, and SW-PSSM (Table 2).

**Table 2:** Performance comparison between the present method (NMF), LSTM, LA-kernel, and SW-PSSM

**Methods**	**Fold level**		**Superfamily level**	
	**Mean ROC**	**Mean ROC**_50_	**Mean ROC**	**Mean ROC**_50_
**NMF**	0.84	0.44	0.96	0.86
**LSTM**	0.70	0.25	0.77	0.39
**LA-kernel**	0.80	0.30	0.88	0.59
**SW-PSSM(2.0, 10, 0.0)**	0.85	0.43	0.96	0.83
**SW-PSSM(3.0,0.75,1.5)**	0.88	0.46	0.96	0.85

LSTM is a fast model-based protein homology detection method without alignment, and LA-kernel is SVM based method using string alignment kernel (please see Availability & requirements below). The best performing method, SW-PSSM is a profile-based local alignment kernel method (please see Availability & requirements below). We measure the ROC and ROC_50 _scores for 34 folds at the fold level and 95 superfamilies at the superfamily level. For the LSTM, we use default parameters and weight (-c lstmpars.mem12.ws12.txt and -w weight.mat) and for the LA-kernel we use version 0.3.2 with β = 0.5 (recommended value). In case of SW-PSSM, we use two parameter sets; default parameter set (gap opening = 2.0, gap extension = 10, zero-shift = 0.0) and another parameter set (gap opening = 3.0, gap extension = 0.75, zero-shift = 1.5) that was reported to be best-performing in the original paper. From Table 2, we note that the performance of SVM output with NMF features is better than the two methods, LSTM and LA-kernel, while SW-PSSM shows slightly better or comparable performance compared to the SVM output with NMF features. It should be noted, however, that our method and the other methods are not directly comparable, and we have not tried to develop better scoring scheme because that is not the main objective of this study. Furthermore, because our method produces more reliable similarity scores between the sequences than conventional PSSM methods, it may be possible to develop a more accurate new kernel-based method using our SVM output scores.

### Variation of performance improvement as a function of the number of positive training examples

**Figure 6:**
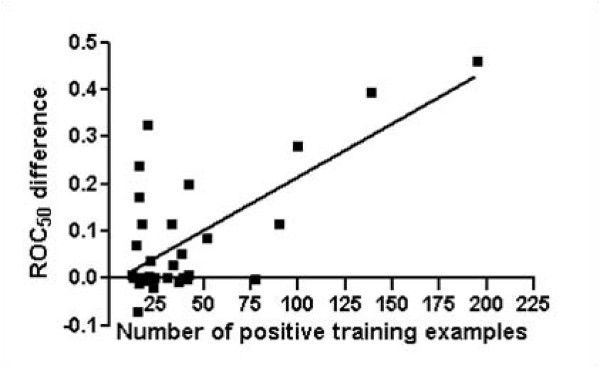
**Variation of performance improvement by using NMF features corresponding to the number of positive training examples**. Figure shows ROC_50 _score improvement for fold recognition at the fold level when NMF features are used, compared to original PPA features. The x-axis indicates the number of positive training examples and y-axis represents performance improvement of mean ROC_50 _scores, respectively.

We investigate ROC_50 _score difference between SVM outputs with NMF features and original PPA results. The figure 6 shows ROC_50 _score improvement when NMF features are used, compared to original PPA features at the fold level. The x-axis indicates the number of positive training examples, and y-axis represents performance improvement of mean ROC_50 _scores at the fold level. The figure indicates that NMF features recognize more homolog proteins than original PPA features in 27 folds among 34 folds. More importantly, the performance improvement is highly correlated with the number of positive training examples. The p-value of correlation slope is 0.0001, indicating that the correlation is very strong. This result is attributed to the NMF property of which NMF can extracts more accurate features when the data set is large. Although at the small number of positive training examples fold level performance improvements look like a random distribution, in fact the correlation becomes stronger as the number of positive training examples increases. We conclude that NMF well recognizes essential features when the number of positive training examples increases.

### Performance variation with number of NMF basis vectors

**Figure 7:**
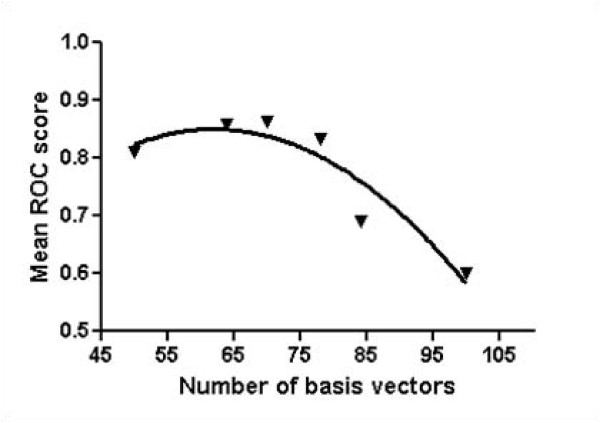
**Mean ROC scores with various number of basis vectors**. The performance of fold recognition depends on the number of NMF basis vectors. On average, the value of 70 is optimal. The mean ROC score of each case is 0.82, 0.85, 0.86, 0.83, 0.69, and 0.60 for 50, 65, 70, 75, 85, and 100 basis vectors, respectively. The optimized number of NMF basis vectors differs in each fold.

In our experiment, a fixed value of 75 is used for the number of NMF basis vectors for each template. However, the number of basis vectors directly determines NMF features. To analyze the effect and find the optimal number of basis vectors, we assess performance variation as a function of the number of basis vectors. First, we divide the training set into two parts, where proteins in each set never share the same superfamily with another. We measure the performance variation of mean ROC scores at the fold level with 6 different values for the number of basis vectors, 50, 65, 70, 75, 85, and 100. The results of mean ROC scores are shown in Figure 7. When the number of basis vectors is 70, the performance seems to be optimized with 0.86 mean ROC score. This experiment remarks that the number of NMF basis vectors significantly affects the performance. Moreover, each fold has a different optimized number of basis vectors.

When we choose the value of 70 as the number of NMF basis vectors, this fixed value may cause a problem for the templates whose sequence lengths are less than 70. Some of NMF basis vectors in those cases can be either duplicated or become zero vectors, possibly leading to performance degradation or improvement for NMF cases. However, the ratio of those events occurring is only 3.2%. Thereby such small effect can be ignored. Furthermore, from our experiments we discover that performance degradation occurs at the templates with a long sequence length rather than at those with a short sequence length. Further investigation is needed to determine the relationship between the number of NMF basis vectors and effective feature extraction.

### NMF basis vectors overlap with functional sites and structurally conserved regions of proteins

Main assumption of this work is that NMF can capture essential features of proteins. In this regard, NMF can reduce redundant regions in each protein, and its basis vectors provide useful information about proteins such as functional sites or structurally conserved regions. To verify our conjecture we conduct the statistical analysis on basis vectors by comparing them with functional sites of proteins and structurally conserved regions.

Due to the part-based representation, NMF basis vectors consist of several blocks of nonzero scores. We compare blocks of NMF basis vectors with functional sites from PROSITE database [29] and structurally conserved regions from multiple structural alignments. To validate NMF's ability to detect functional sites and structurally conserved regions, we make two types of random basis vectors. 'Block random basis vector' is a vector in which nonzero *blocks *are randomly re-distributed along the vector, whereas 'point random basis vector' is a vector in which nonzero *scores *are randomly re-distributed. We also create 'PPA vectors', which are composed of the top 5% PPA scores.

**Figure 8:**
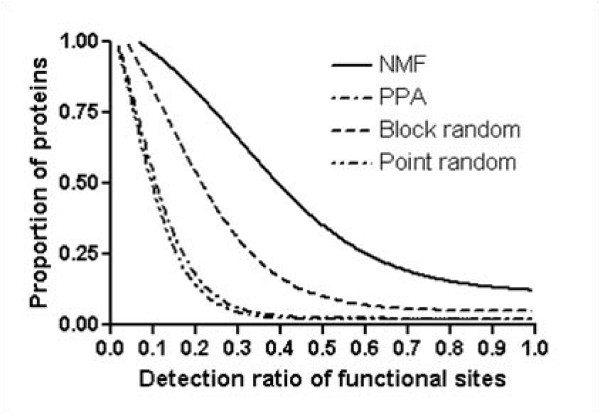
**Proportion of proteins corresponding to given functional sites coverage**. This figure shows functional coverage variation of NMF, original PPA, block random, and point random basis vectors. The x-axis represents the detection ratio of functional sites, the y-axis the proportion of proteins. ScanProsite gives predicted functional sites. NMF and PPA denote the NMF basis vectors and original PPA vectors. Block (Point) random basis vector are build by rearrange blocks (points) in NMF basis vectors. Figure shows that NMF basis vectors match with functional sites more than other vectors.

First we evaluate the ability of NMF basis vectors to detect functionally important sites of each protein by comparing them with the functional sites of proteins from PROSITE. We use ScanProsite, which allows us to scan protein sequences for patterns of functional sites stored in the PROSITE database [29]. Figure 8 shows the proportion of proteins as a function of the overlap ratio of NMF basis vectors with functional sites, along with those of PPA, block random basis vectors, and point random basis vectors. It is clear that NMF basis vectors detect a significantly larger number of functional sites than PPA vectors, block random basis vectors, or point random basis vectors. The average number of functional sites in 3118 proteins is 10. On average, NMF basis vectors detect 3.2 functional sites, while PPA vectors, block random basis vectors, and point random basis vectors detect 0.9, 1.7, and 0.8 functional sites, respectively. Furthermore, the proportion of proteins from which more than 50% of functional sites are detected is 36%, 2.2%, 10%, and 2.2% for NMF basis vectors, PPA vectors, block random basis vectors, and point random basis vectors, respectively. These results remark that NMF is capable of extracting functionally important sites of proteins.

**Figure 9:**
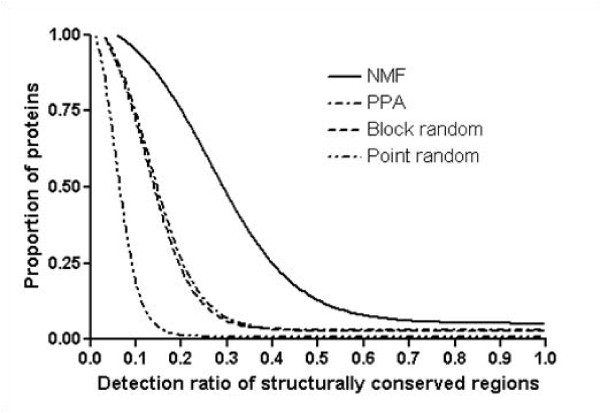
**Proportion of proteins corresponding to given structurally conserved sites coverage**. This figure shows structural coverage variation of NMF, original PPA, block random, and point random basis vectors. The x-axis represents the detection ratio of structurally conserved regions, and the y-axis the proportion of proteins. MUSTANG is used for multiple structural alignments, from which we define structurally conserved regions as regions that are aligned for more than 95% Figure indicates that NMF basis vectors match with structurally conserved regions more than other vectors.

Next, to verify NMF's ability to recognize evolutionary conserved regions from a structural perspective, we compare NMF basis vectors with the structurally conserved regions from multiple structural alignments. Structurally conserved regions are important, as they maintain the structural features which define characteristics to a given fold. We use MUSTANG_v.3 [30] for multiple structural alignments, and structurally conserved regions are defined as over 95% conserved residues. Figure 9 shows the proportion of proteins as a function of given structural coverage variation of NMF basis vectors, PPA vectors, block random basis vectors, and point random basis vectors. The average number of structurally conserved regions in 63 folds is 6.7. On average, NMF basis vectors detect 1.7 structurally conserved regions in 63 folds, whereas PPA vectors, block random basis vectors, and point random basis vectors detect 0.6, 0.8, and 0.4 structurally conserved regions, respectively. Furthermore, the proportion of proteins for which more than 50% of functional sites detected is 14%, 3.0%, 3.0%, and virtually 0% for NMF, PPA, block random, and point random, respectively. This result verifies that NMF basis vectors effectively represent significant portions of structurally conserved regions.

From the two statistical analyses on functional sites and structurally conserved regions, we note that NMF basis vectors represent essential parts of sequences. SVM's ability to recognize remote homologs depends on feature vectors. SVMs are trained well if feature vectors consist only of essential features for remote homologs detection. In this regard, NMF reconstructs original PPA feature vectors with noise reduction and preserves core regions such as functionally important sites and structurally conserved regions. Therefore, NMF improves the performance of fold recognition.

## Conclusion

In this work, we investigate the possibility of applying NMF to improve the profile-profile alignment features for fold recognition and remote homolog detection. We show that when NMF feature extraction method is used, the performance is greatly improved, compared to previous fold recognition algorithms. The main reason for the improvement is that NMF feature extraction method reduces "noises" and extracts essential features from PPAs. Due to this noise reduction property, SVM with NMF features shows a great performance improvement for fold recognition at the fold level and the remote homolog detection at the superfamily level. We also find that improvement is bigger when data set is larger, and the number of basis vectors needs to be optimized for the best performance. As an evidence for NMF's ability to extract the essential features from sequences, we discover that there exists a close relationship between NMF basis vectors and functional sites or structurally conserved regions of proteins. This supports our conjecture that NMF basis vectors explicitly represent essential features of proteins.

Feature extraction using NMF gives us intuitive understanding about the feature vectors. We can extract more useful feature vectors and analyze them to better understand the biological meaning of protein sequences, which makes NMF feature extraction method a promising tool not only for the fold recognition but also for the analysis of large-scale biological data. Furthermore, as we point out in Result Section, our method produces more accurate similarity scores between the sequences than conventional PSSM methods, which would allow us to develop more accurate kernel-based method based on our method.

For the future work we can use NMF to improve alignment quality. In fold recognition problem, improvement in sequence alignment accuracy remains a challenge, as existing methods still do not always reach the level of the best alignment possible [31]. Accurate sequence alignments undoubtedly increase the performance of fold recognition. Our results indicate that NMF methods can remove false alignments by allowing only a combination of essential features in profile-profile alignment. In this regards, we believe that NMF can be a promising method to improve sequence alignment accuracy. Furthermore, by analyzing NMF basis vectors, we can extract intuitive information from protein sequences, which may be used for motif search.

## Methods

### Data

We construct the template library based on SCOP ASTRAL Compendium version 1.67 [32]. Proteins in the library share less than 40% sequence identity with each other. The domains in the classes a, b, c, d, and e are used, and discontinuous domains are removed. For fold recognition we randomly divide all templates into training set (2437 proteins) and testing set (630 proteins and 34 folds), where they do not come from the same superfamily. In this setting, protein domains within the same fold are considered as positive training examples. For remote homolog detection we also randomly divide all templates into training set (2342 proteins) and testing set (435 proteins and 94 superfamilies), they do not share the same family members. For both fold recognition and remote homolog detection, protein domains in outside the same fold are considered as negative examples.

### A profile-profile alignment feature vectors and feature extraction with NMF

To build NMF feature vectors for SVMs in the training set, we first generate all-against-all alignments by profile-profile alignment scheme, without using any structural information [21]. The profile of a sequence **X **of length *n *is represented by two *n *× 20 matrices. The first matrix is a position-specific scoring matrix (PSSM) of the sequence, which is computed directly by PSI-BLAST. We use blastpgp version 2.2.15 with default parameters except for the number of iterations (j = 11) and cutoff value of e-value (h = 0.001). The rows of PSSM matrix correspond to the residues in **X **and the columns correspond to the 20 distinct amino acids. The second matrix is a position-specific frequency matrix, which contains the frequency occurrences of 20 amino acids at each column in the multiple sequence alignement. The profile-profile alignment score for aligning the position *i *of a template *q *and the position *j *of a template *t *is given by

(1) $$m_{ij}=\sum_{k=1}^{20}\left[f_{ik}^{q}S_{jk}^{t}+S_{ik}^{q}f_{jk}^{t}\right]$$

where $f_{ik}^{q}$, $f_{jk}^{t}$, $S_{ik}^{q}$, and $S_{jk}^{t}$ are the frequencies (i.e. $f_{ik}^{q}$ and $f_{jk}^{t}$) and the PSSM scores (i.e. $S_{ik}^{q}$ and $S_{jk}^{t}$) of amino acid *k*, at position *i *of a template *q *and position *j *of a template *t*, respectively. If gaps occur, fixed negative scores are assigned. For each template of length *n *in the training set, alignments with the other templates in the training set are generated. Then, these alignments are transformed, respectively, into (*n*+2)-dimensional feature vectors, (*sa*_1_, *sa*_2_, *sa*_3_, ..., *sa*_*n*_, total_score, sequence_length), where *sa*_*i*_, total_score, and sequence_length are a profile-profile alignment score at position *i *of the template, total profile-profile alignment score, the length of the template, *i.e., n*, respectively. Total score and sequence length are normalized when SVM is trained.

Next, NMF is applied to this feature vectors to build the NMF feature vectors by performing matrix factorization. At the matrix factorization step, the total score and sequence length in the original feature vectors are excluded (therefore *n*-dimensiona1 feature vectors), and then later added to the NMF feature vectors. All *n*-dimensional feature vectors are placed in the columns of *n *× *m *matrix **V **where *m *is the number of training examples in the dataset. Given a nonnegative matrix **V**, we find nonnegative matrix factors **W **and **H **such that [1]:

(2) **V **≈ **W H**

**Figure 10:**
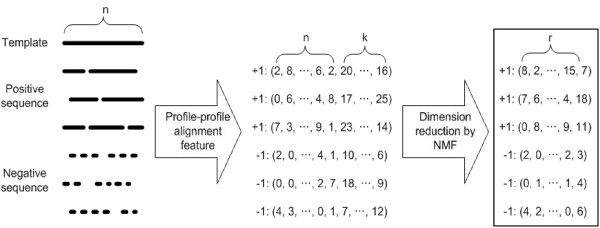
**The whole procedure of feature extraction**. Nonnegative matrix factorization (NMF) is used for part-based sequence representation. The template sequence of length *n *is aligned to the sequences of positive (solid line) and negative (dot line) examples by profile–profile alignment method. Next, each alignment is transformed to (*n *+ 2) -dimensional feature vector that is composed of the alignment scores at *n *positions, the total alignment score and sequence length. Finally, NMF is applied to feature vector for all except two features: total alignment score and sequence length. These extracted feature vectors are used to train SVM for a target template.

The matrix is then approximately factorized into *n *× *r *matrix **W **and *r *× *m *matrix **H**, where **W **is a set of *r *basis vectors and **H **is a set of coefficient vectors for *m *training examples. Before NMF, We need to transform the *n*-dimensional feature vectors using a sigmoid function, $\frac{1}{1+e^{-sa_{\text{i}}}}$, to make the matrix **V **nonnegative The sigmoid function changes the range of original PPA feature vectors from [-5 5] to [0 1]. Transforming feature vectors with a sigmoid function shows better result than adding the constant bias score 5 to the *n*-dimensional feature vectors, indicating that sigmoid function is more appropriate for preserving the original feature vector space. NMF is conducted using recently proposed projected gradient method [33] instead of the original multiplicative learning rule for faster convergence. We add two features to *r*-dimensional nonnegative feature vectors, where two features are the total score and sequence length which are normalized to have zero mean and unit variance. Thereby, the final feature vector is (*r*+2) dimensional coefficient vector. Figure 10 summarizes the modified procedure of feature extraction using NMF.

In our experiment, a fixed value of 70 is used for the number of NMF basis vectors for each template. This value is optimized from experiments. We divide the training set into parameter training set (1660) and parameter testing set (426), where proteins in each set never share the same superfamily with another. We assess the performance of mean ROC score at the fold level with various numbers of basis vectors, 50, 65, 70, 75, 85, and 100. From the figure 7, we consider the value of 70 as an optimized number of basis vectors in our experiment.

### SVM training

SVM is implemented by using SVM-light (please see Availability & requirements below), freely available SVM software, with the radial basis function as a kernel, $k(x,x')=e^{-\gamma {\left(x-x'\right)}^{2}}$. We use default option for SVM except that the value of γ is fixed to 0.005 for the original PPA as in the previous work, and 0.0055 is chosen for the NMF features after trying several values of γ (0.001, 0.005, 0.0055, 0.01, 0.05, and 0.1). For each SVM output we add mean output scores of SVM machines which are included in the same fold. This modification reduces variance of SVM outputs, leading to stabilize the performance of SVM machines.

### Testing and performance assessment

We generate the profile-profile alignments between the test proteins in the testing set and the templates in the training set, and transform them to the feature vectors, which are then evaluated by the trained SVMs to produce outputs. Fold recognition performances are measured by the receiver operating characteristic (ROC) scores and the ROC_50 _scores. ROC score is defined as the areas under the ROC curves, the plot of true positives as a function of the number of false positives [34]. At the fold level, proteins in the same fold, but different superfamily are identified as homologs. At the superfamily level, proteins in the same superfamily but different family are considered as homologs. Otherwise, proteins in the different folds are defined as non homologs.

### Comparison of NMF basis vectors with functionally important sites and structurally conserved regions

To define functional sites, we use ScanProsite, which provides predicted functional sites by scanning protein sequences for patterns of functional sites stored in the PROSITE database [29]. We use MUSTANG_v.3 [30] for multiple structural alignments and define over 95% structurally conserved regions in each fold as structurally important sites.

If we apply NMF to all the alignments between a query and the proteins in both the positive and negative sets, it becomes difficult to decide which basis vectors represent the biologically meaningful features of the query protein. Therefore, for meaningful analysis of NMF basis vectors, we extract the basis vectors by applying NMF only to alignments with sequences in the positive set. We create the positive set not only from the training set but also from testing set. As a result, there are 3118 new alignments, from which new NMF basis vectors are calculated.

We use top 5% values of nonzero scores in basis vectors to eliminate outlier scores. We assume that if the corresponding portion of a functional region or structural region with a block is more than a cutoff value of 50%, the functional region or structural region is considered to be matched. We find that the choice of the cutoff value is not very critical. For example, when we change the cutoff value from 50% to 65%, the number of matched regions remains virtually the same. To validate NMF's ability to match biologically meaningful regions, we create two types of basis vectors for comparison. The first type of basis vector is called 'PPA vectors', which are composed of the top 5% of raw PPA scores. The other type of basis vectors are two different kinds of random basis vectors: 'block random basis vector' and 'point random basis vector.' In 'block random basis vector', nonzero *blocks *of the NMF feature vectors are randomly re-distributed, whereas in 'point random basis vector', all nonzero *scores *of the NMF feature vectors are randomly re-distributed. The reason for 'block random basis vectors' is due to unique structural property of NMF feature vectors; they typically consist of several blocks of nonzero scores. Therefore, it is more meaningful to compare NMF feature vectors with 'block random basis vectors'. As shown in Figure 8 and Figure 9, 'point random basis vectors' have fewest overlaps with functional sites or structurally conserved regions, since the functional motif or structurally conservation sites are usually present as blocks.

## Availability & requirements

LSTM is downloaded from: 

LA-kernel is downloaded from: 

SW-PSSM is downloaded from: 

SVM-light is downloaded from: 

## Authors' contributions

IJ, JL, S–YL, and DK designed the methods, and experimental setup. IJ carried out the implementation of the various methods except a NMF algorithm, and JL implemented a NMF algorithm. IJ and DK wrote the manuscript. All authors have read and approved the final manuscript.
